# Neural substrates of perception in the vestibular thalamus during natural self-motion: A review

**DOI:** 10.1016/j.crneur.2023.100073

**Published:** 2023-01-11

**Authors:** Kathleen E. Cullen, Maurice J. Chacron

**Affiliations:** a Department of Biomedical Engineering, Johns Hopkins University, Baltimore, USA; b Department of Otolaryngology-Head and Neck Surgery, Johns Hopkins University School of Medicine, Baltimore, USA; c Department of Neuroscience, Johns Hopkins University School of Medicine, Baltimore, USA; d Kavli Neuroscience Discovery Institute, Johns Hopkins University, Baltimore, USA; e Department of Physiology, McGill University, Montréal, Canada

**Keywords:** Vestibular, Adaptation, Optimal coding, Neural coding, Perception, Voluntary movement

## Abstract

Accumulating evidence across multiple sensory modalities suggests that the thalamus does not simply relay information from the periphery to the cortex. Here we review recent findings showing that vestibular neurons within the ventral posteriolateral area of the thalamus perform nonlinear transformations on their afferent input that determine our subjective awareness of motion. Specifically, these neurons provide a substrate for previous psychophysical observations that perceptual discrimination thresholds are much better than predictions from Weber's law. This is because neural discrimination thresholds, which are determined from both variability and sensitivity, initially increase but then saturate with increasing stimulus amplitude, thereby matching the previously observed dependency of perceptual self-motion discrimination thresholds. Moreover, neural response dynamics give rise to unambiguous and optimized encoding of natural but not artificial stimuli. Finally, vestibular thalamic neurons selectively encode passively applied motion when occurring concurrently with voluntary (i.e., active) movements. Taken together, these results show that the vestibular thalamus plays an essential role towards generating motion perception as well as shaping our vestibular sense of agency that is not simply inherited from afferent input.

## Highlights

• Vestibular thalamocortical neurons do not merely relay information from the periphery to the cortex.
• Nonlinear response properties provide a substrate for observed deviations from Weber's law in motion perception.
• These nonlinearities allow for optimal and unambiguous encoding of natural but not artificial stimuli.
• Vestibular thalamocortical neurons mediate our sense of agency by selectively encoding passive motion over voluntary movements.
• Nonlinear operations performed byhe vestibular thalamus play an essential role towards shaping our generating motion perception of self-motion.

## Graphical abstract

**Figure:**
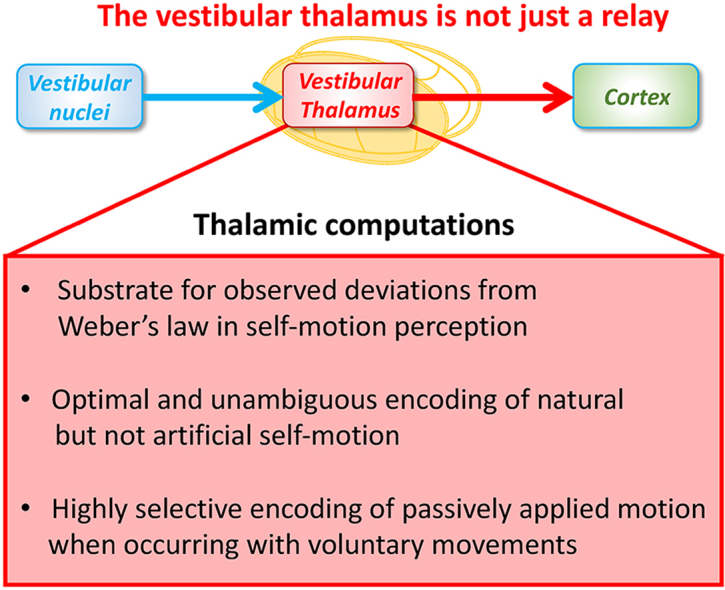

## 1 Introduction

Understanding how neurons represent sensory information to generate perception and behavior remains an important problem in systems neuroscience. Towards this goal, the functional role of the thalamus remains the matter of debate. A long-standing view has been that the thalamus effectively serves as a passive relay that faithfully transmits sensory information from the periphery to the cortex for further processing (see (Sherman and Guillery, 2002; Sherman and Guillery, 2006; Sherman, 2007) for review). More recent neurophysiological and lesion studies in alert behaving animals have provided accumulating evidence that the thalamus instead plays an active role in shaping the representation of sensory information that we experience in our everyday lives (see (Mitchell et al., 2014) for review). To date, most of these studies have been carried out in visual, auditory, and somatosensory areas of the thalamus (Wurtz et al., 2011; Bartlett, 2013; Buchan et al., 2021). As such, comparatively little is known about how the thalamus processes vestibular information. Accordingly, here we review recent results showing that vestibular thalamic neurons do far more than passively relay information to cortex. Rather, they perform nonlinear operations on their input that shape our perception of self-motion.

## 2 Ascending vestibular thalamic pathways

**Fig. 1:**
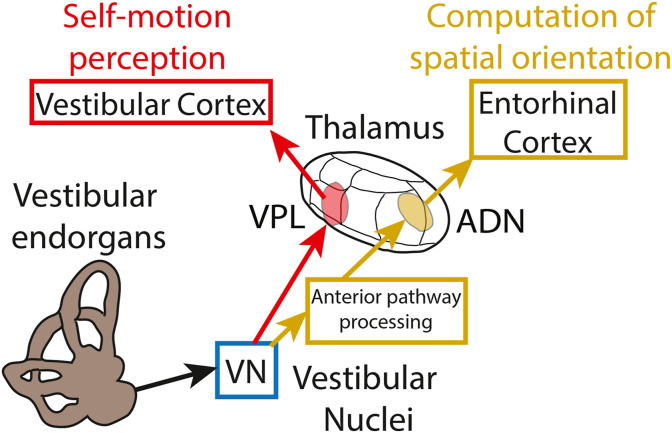
Overview showing brain areas and functional roles of the two major ascending vestibular pathways. Vestibular afferents innervating the vestibular endorgans project to the vestibular nuclei (VN). VN neurons in turn project directly to the ventral posteriolateral (VPL) area of the thalamus and indirectly to the anterodorsal thalamic nucleus (ADN) via multiple brain areas (see “Anterior pathway processing”). While VPL neurons project to vestibular cortical areas that mediate self-motion perception. ADN neurons instead project to other areas such as the entorhinal and retrosplenial cortices that mediate computation of spatial orientation and are reviewed elsewhere (Cullen and Taube, 2017). Detailed diagrams of both pathways have been published previously (Cullen and Taube, 2017; Lopez and Blanke, 2011; Clark and Harvey, 2016). Abbreviations: VN: vestibular nuclei; VPL: ventral posteriolateral; ADN: anterodorsal thalamic nucleus.

The vestibular system not only plays a critical role in mediating vital reflexes to stabilize gaze and posture but also makes essential contributions towards the higher-level computations required for the perception of spatial orientation and self-motion. Afferents innervating the vestibular endorgans project to neurons within the vestibular nuclei, which in turn project to the thalamus (Fig. 1). There are two major vestibular thalamic pathways, one anterior (Fig. 1, gold) and one posterior (Fig. 1, red), that are thought to transmit vestibular information from the thalamus to the cortex. The anterior pathway is an essential component of the head direction cell network. It comprises projections from the anterodorsal thalamic nucleus (ADN) to the retrosplenial and entorhinal cortices (Fig. 1, upper right). This pathway has been well studied and is thought to provide a neural representation of heading direction during movement that is critical for navigation (see (Cullen and Taube, 2017; Lopez and Blanke, 2011) for review). The posterior pathway, which is the focus of the current review, involves direct projections from vestibular nuclei neurons to neurons within the ventral posteriolateral area (VPL) of the thalamus that in turn project to vestibular cortical areas (Fig. 1, upper left). This pathway, which has been less extensively studied, is thought to process vestibular information in order to compute essential representations of self-motion and spatial orientation required for ensuring the accurate coordination of perception and action (see (Clark and Harvey, 2016) for review).

Neurons within VPL can respond to multiple modalities including proprioception, tactile, and vestibular (see (Lopez and Blanke, 2011) for review). VPL neurons that are sensitive to vestibular, henceforth referred to as vestibular thalamocortical neurons, receive direct inputs from the vestibular nuclei (Marlinski and McCrea, 2009) as well as deep cerebellar nuclei (Meng et al., 2007). Neurons in the vestibular nuclei that project to the VPL respond to passive sinusoidal rotations around the yaw axis (i.e., activation of the horizontal canals, which are roughly parallel to the floor) and/or translations in the horizontal plane and are insensitive to eye movements. Thus, they correspond to a population of neurons commonly referred to as vestibular-only neurons in the literature (reviewed in (Cullen, 2019)). Notably, this population is distinct from other neural populations within the vestibular nuclei that mediate the vestibular ocular reflex (see (Cullen, 2012) for review). Notably, vestibular-only neurons also send projections to the spinal cord to mediate vestibulo-spinal reflexes (Goldberg et al., 1987; Sato and Sasaki, 1993). The direct projection from the vestibular-only neurons within the vestibular nuclei to vestibular thalamocortical neurons raises the questions: do vestibular thalamocortical neurons merely relay information encoded by vestibular nuclei neurons to the cortex? Or does the vestibular thalamic pathway instead extract certain features that contribute to higher level processing?

## 3 Vestibular thalamocortical neurons provide a neural substrate for perceptual performance

Previous studies in human subjects have shown that vestibular perceptual performance is actually not consistent with Weber's Law (Mallery et al., 2010). Weber's law, which has been observed in a variety of sensory modalities, states that the discrimination threshold or “just noticeable difference” increases linearly as a function of stimulus amplitude (Weber et al., 1834). Instead, human vestibular discrimination thresholds demonstrate an initial linear increase with increasing stimulus amplitude but then saturate for higher amplitude stimulation. This saturation implies that self-motion perceptual discrimination thresholds are actually considerably better (i.e., lower) than those predicted by Weber's law for higher stimulus amplitudes.

**Fig. 2:**
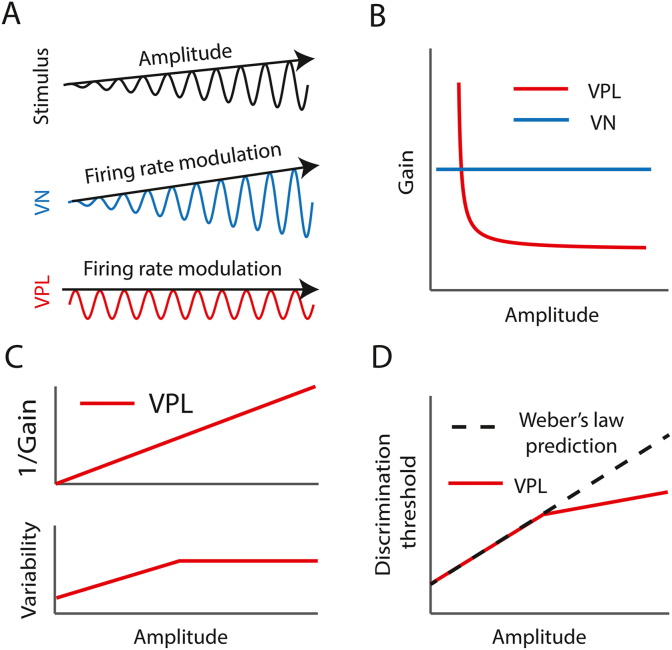
A) *Top:* Self-motion stimulus consisting of a sinewave whose amplitude increases linearly with time. *Middle*: VN response to this stimulus. *Bottom:* VPL response to this stimulus. Note that, while VN neurons respond with changes in firing rate whose amplitude increases with time, VPL neurons display contrast gain control, such that the amplitude modulation in their responses remains constant. **B)** Gain of VN (blue) and VPL (red) responses as a function of stimulus amplitude. While VN neural gain is constant, VPL neural gain decreases as a power law with increasing amplitude. **C)***Top:* The inverse gain increases linearly with increasing amplitude for VPL. *Bottom:* Neural variability in VPL neurons first increases and then saturates with increasing amplitude. **D)** VPL neural discrimination threshold as a function of amplitude which is obtained by dividing the neural variability by the gain. The fact that neural variability saturates for larger amplitudes causes a deviation from Weber's law (dashed line), which qualitatively agrees with psychophysical studies (Mallery et al., 2010). (For interpretation of the references to colour in this figure legend, the reader is referred to the Web version of this article.)

This then raises the more specific question: How does the vestibular thalamus contribute to the observed violations of Weber's law observed in human self-motion perception? A recent study conducted in non-human primates provides new insights that directly address this question (Carriot et al., 2021). The activity of individual vestibular thalamocortical neurons were recorded while vestibular stimulation was varied over a wide range of amplitudes. Quantification of neuronal responses revealed that modulation gains decreased as a power law with increasing stimulus amplitude (Fig. 2A), consistent with the results of prior studies (Marlinski and McCrea, 2008a; Dale and Cullen, 2019). The observed decrease in gain with increasing stimulation amplitude is indicative of ‘contrast gain control’ (Fig. 2B). The prevailing view is that contrast gain control ensures that the firing rate remains within the dynamic range (Marlinski and McCrea, 2008a) and has been reported in both subcortical and cortical brain areas associated with processing sensory information from other modalities (Rabinowitz et al., 2011; Lohse et al., 2020; Maravall et al., 2007; Carandini and Heeger, 2012; Carandini et al., 1997, 2002).

To understand how contrast gain control displayed by vestibular thalamocortical neurons influences perceptual discrimination, neural discrimination thresholds were computed and compared to perceptual discrimination thresholds. Neural discrimination thresholds are influenced by neural sensitivity (i.e., gain), such that increased sensitivity leads to a lower threshold (i.e., better discrimination). However, such thresholds are also influenced by variability. This is because increased variability will lead to an increased threshold (i.e., worse discrimination). As such, neural discrimination thresholds are proportional to the variability-to-gain ratio (Green and Swets, 1966). Because, as mentioned above, gain decreases as a power law with increasing amplitude (Fig. 2B, red), the inverse gain increases linearly (Fig. 2C, upper panel). Further, quantification of neural variability revealed an initial increase followed by saturation with increasing stimulus amplitude (Fig. 2C, lower panel). As a result, neural discrimination thresholds initially increased linearly with stimulus amplitude, consistent with predictions from Weber's law, but increased more slowly at higher amplitudes, thereby deviating from predicted values (Fig. 2D). Thus, the dependencies of gain and variability on stimulus amplitude in vestibular thalamocortical neurons can account for why self-motion perceptual discrimination thresholds are considerably better than predictions from Weber's law (Carriot et al., 2021).

## 4 Vestibular thalamocortical neurons show reduced coding ambiguity during natural stimulation

**Fig. 3:**
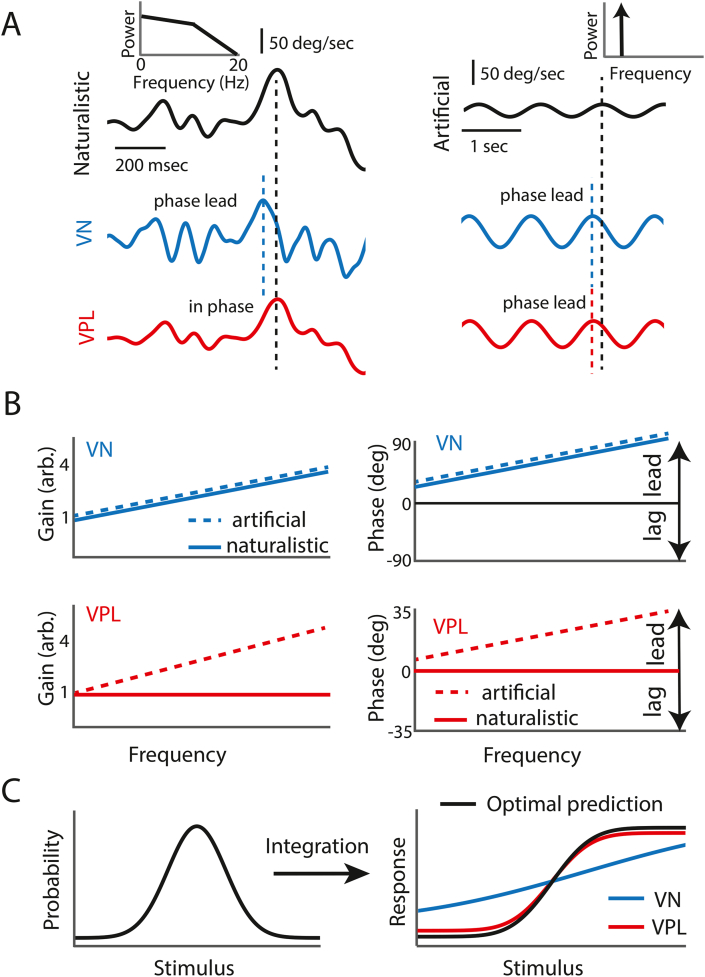
A) *Top:* Naturalistic (left) and artificial (right) time series. Note that naturalistic stimuli can reach much higher amplitudes than the artificial sinusoidal stimuli that have been typically used. Importantly, naturalistic stimuli contain a spectrum of frequencies (left inset) whereas sinusoidal stimuli only contain one frequency (right inset). *Middle*: VN responses (blue) to naturalistic (left) and artificial (right) stimulation. Note that, in both cases, the response leads (i.e., reaches its maximum before) the stimulus (dashed lines and blue arrows). *Bottom:* VPL responses to naturalistic (left) and artificial (right) stimulation. Note the important difference that, under naturalistic stimulation, VPL responses faithfully follow the stimulus waveform with no significant phase lead, whereas phase leads similar to those observed for VN are seen under artificial stimulation (dashed lines and red arrows). **B)***Top left:* Gain increases with increasing frequency for VN during both artificial (dashed blue) and naturalistic (solid blue) stimulation. Note that both curves overlap and are offset here for clarity. *Top right*: Phase lead increases with increasing frequency for VN during artificial (dashed blue) and naturalistic (solid blue) stimulation. Note that both curves overlap and are offset here for clarity. *Bottom left:* While gain increases with increasing frequency for VPL during artificial stimulation (dashed red), gain was instead independent of frequency for VPL during naturalistic stimulation (solid red). *Bottom right*: While phase lead increases with increasing frequency for VPL during artificial (dashed red), no phase lead is seen during naturalistic stimulation (solid red). **C)** Optimal coding theory predicts that the neural tuning curve (right panel, black curve) should be related to the stimulus probability distribution (left panel, black curve) through integration. The actual tuning curve for VPL (right panel, red curve) better matches the optimal prediction (right panel, black curve) than the actual tuning curve for VN (right panel, blue curve). This indicates that VPL neurons are better optimized to encode naturalistic stimuli than VN neurons. (For interpretation of the references to colour in this figure legend, the reader is referred to the Web version of this article.)

The results described above are interesting in that they provide a neural correlate (Carriot et al., 2021) for human perceptual thresholds measured during psychophysical studies performed in a laboratory setting (Mallery et al., 2010). However, because the stimuli applied in this recent electrophysiological study varied sinusoidally, the experimental design could not address how vestibular thalamocortical neurons encode the more complex head movements that are experienced during everyday activities. Natural stimuli across modalities in general display complex time-varying spatiotemporal characteristics and thus strongly differ from the artificial stimuli that are typically used to study neural responses (Field, 1987; Attias and Schreiner, 1997; Simoncelli and Olshausen, 2001; Lewicki, 2002; Fotowat et al., 2013). In the case of the vestibular system, behavioral studies have quantified the statistics of self-motion experienced by both human and non-human primates during everyday activities. Overall, natural vestibular stimuli fundamentally differ from the artificial stimuli (e.g., sinusoids) typically used (Carriot et al., 2014, 2017) (see (Cullen, 2019) for review). Specifically, natural vestibular stimuli reach much higher amplitudes than artificial stimuli (Fig. 3A, top left and right panels) and further display spectral power that decays with increasing frequency over the natural range of self-motion (0–20 Hz) as opposed to comprising a single frequency (see (Cullen, 2019) for review) (Fig. 3A, compare top left and right insets). Importantly, growing evidence from other modalities shows that sensory systems have adapted their coding strategies to the statistics of natural stimuli, emphasizing their importance when studying sensory coding (Laughlin, 1981; Dan et al., 1996; Attneave, 1954; Barlow and Rosenblith, 1961; Olshausen and Field, 2004; Wark et al., 2007; Huang and Chacron, 2016; Huang et al., 2016). In the case of the vestibular system, recent neurophysiological investigations have likewise shown that the responses of both afferents and vestibular nuclei neurons are adapted to the statistics of natural vestibular stimulation (Schneider et al., 2015; Mitchell et al., 2018; Mackrous et al., 2020). To date, vestibular thalamocortical neurons have primarily only been recorded during artificial stimulation and were found to display response dynamics that are generally comparable to those of vestibular nuclei neurons (Meng et al., 2007; Meng and Angelaki, 2010; Marlinski and McCrea, 2008b). This then raises the question of how does the vestibular thalamus encode natural stimuli?

A recent study addressed this question by recording the responses of individual vestibular nuclei and their target vestibular thalamocortical neurons during naturalistic (Fig. 3A, upper left black curve) and artificial (Fig. 3A, upper right black curve) stimulation consisting of rotations within the horizontal plane. It was found that vestibular nuclei neurons largely show comparable response dynamics during both stimulation protocols in that the neural response led (i.e., reaches its maximum before) the stimulus (Fig. 3A, compare blue and black curves). Indeed, quantifying response gain and phase revealed similar dependencies on frequency (Fig. 3B, upper panels). Specifically, response gains during both stimulation paradigms similarly increased as a function of frequency (Fig. 3B, upper left panel, compare solid and dashed blue). In contrast, vestibular thalamocortical neurons differentially encoded natural and artificial stimulation (Fig. 3A, compare red curves). Specifically, response gain to artificial stimuli increased with frequency in a manner similar to that shown for vestibular nuclei neurons above (Fig. 3B, compare dashed red in lower left panel to solid and dashed blue in upper left panel), while those to natural stimulation were instead strikingly constant as a function of frequency (Fig. 3B, lower left panel, solid red). In addition, thalamic neuronal response phases differed from those of vestibular nuclei neurons under naturalistic but not artificial stimulation. Specifically, vestibular nuclei neurons showed marked phase leads that increased with respect to head velocity for both naturalistic and artificial self-motion stimulation (Fig. 3B, upper right panel, compare solid and dashed blue). In contrast, vestibular thalamocortical neurons showed similar phase leads as vestibular nuclei neurons to artificial stimuli but no significant phase leads to naturalistic stimuli (Fig. 3B, flower right panel, compare solid and dashed red). As a result, the responses of neurons were effectively in phase with head velocity.

Importantly, the phase relationship displayed by vestibular thalamocortical neurons during naturalistic stimulation leads to a one-to-one relationship between input stimulus (e.g., velocity) and the output firing rate. In contrast, the phase leads displayed by vestibular nuclei neurons during both artificial and naturalistic stimulation as well as those displayed by vestibular thalamocortical neurons during artificial stimulation led to coding ambiguity. This is because there is then no one-to-one relationship between input stimulus (e.g., velocity) and the output firing rate; a given value of the head velocity elicits different firing rates (Carriot et al., 2022). On the other hand, the phase leads observed in the vestibular nuclei, while giving rise to ambiguity, are thought to be beneficial for motor control. Specifically, dynamic phase leads are required by vestibulo-spinal reflex pathways to compensate for the high inertia of the head-neck system (Fernandez and Goldberg, 1971; Bilotto et al., 1982) (see (Cullen, 2019) for review). Overall, the findings described above establish: 1) that vestibular thalamocortical neurons unambiguously encode instantaneous head velocity during naturalistic but not artificial stimulation and 2) that this fundamental change in response dynamics is not trivially inherited from vestibular nuclei neurons. Moreover, a phenomenological model including contrast gain control (Dean et al., 2005; Butts et al., 2007) can explain these findings (Carriot et al., 2022).

To then address whether vestibular thalamocortical neurons were better adapted to the statistics of natural self-motion stimuli than vestibular nuclei neurons, additional analyses were performed based on optimal coding theory, which is based on the hypothesis that sensory systems use strategies that take advantage of the statistical structure of stimuli that are encountered in the natural environment such as to optimally represent them (Wark et al., 2007). While this theory can take many forms, perhaps the simplest posits is that coding optimized (i.e., the amount of information transmitted about the stimulus is maximum) when the neuronal tuning curve (i.e., the relationship between input stimulus and output firing rate) is proportional to the cumulative integral of the stimulus probability distribution (Laughlin, 1981; Wark et al., 2007) (Fig. 3C). For example, if the stimulus probability distribution is Gaussian, then the optimal tuning curve is a sigmoid (Fig. 3C). Intuitively, such a relationship between the neuronal tuning curve and the stimulus probability distribution makes sense as stimuli with the highest probability of occurrence give rise to neural responses with the highest gain, which maximizes discriminability. Since natural vestibular stimuli display a bell-shaped probability distribution (Carriot et al., 2014, 2017; Schneider et al., 2015) (Fig. 3C, left panel, black), the tuning curve that optimizes information transmission has a sigmoidal shape (Fig. 3C, right panel, black). Comparing experimentally obtained tuning curves to that predicted from optimal coding theory revealed a much better match for vestibular thalamocortical neurons than for vestibular nuclei neurons (Fig. 3C, right panel, compare red and blue curves with black curve). This result was observed irrespective of how self-motion is represented (e.g., position, velocity, acceleration, etc …) (Carriot et al., 2022). Thus, vestibular thalamocortical neurons also more optimally encode natural self-motion stimuli than their input vestibular nuclei neurons.

## 5 Vestibular thalamocortical neurons more selectively encode the passive component of self-motion than vestibular nuclei neurons

**Fig. 4:**
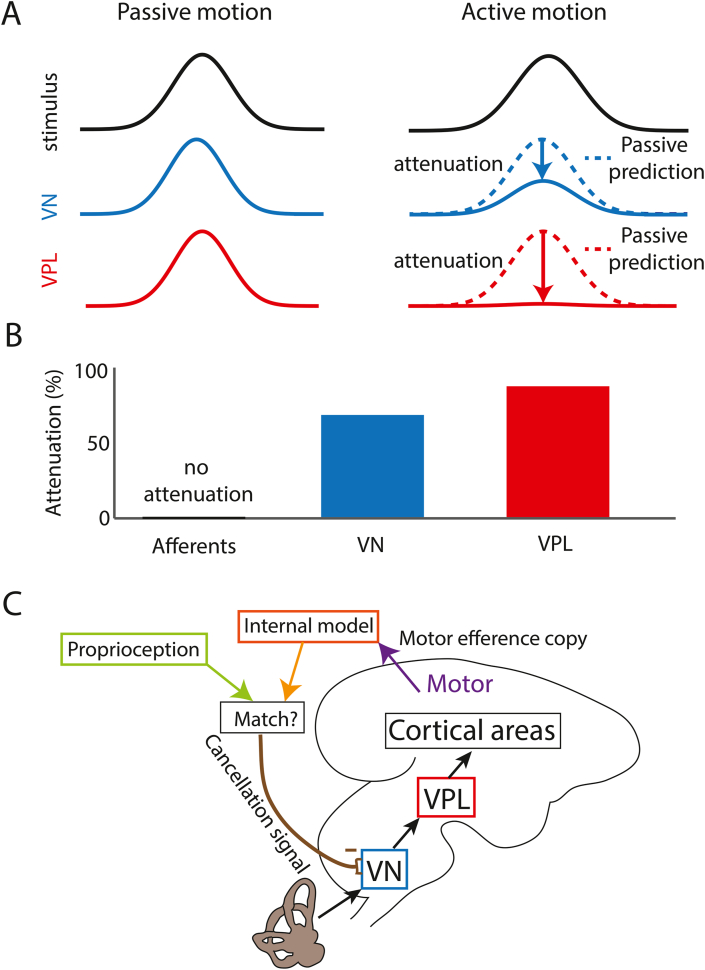
A) *Top:* Stimulus waveforms during passive (left) and active (right) motion. *Middle*: VN responses during passive (left) and active (right) motion. Note that responses are attenuated during active motion, as can be ascertained by comparing the actual response (solid blue) to that predicted from passive motion (dashed blue) and is indicated by the blue arrow. *Bottom:* VPL responses during passive (left) and active (right) motion. Note that responses are attenuated during active motion, as can be ascertained by comparing the actual response (solid red) to that predicted from passive motion (dashed red) and is indicated by the red arrow. Note that VPL responses are in general more attenuated than those of VN. **B)** Bar graph showing % attenuation during active motion for vestibular afferents (left), VN (middle), and VPL (right). Note that afferent responses are not attenuated and that the bar is offset with respect to the x-axis for clarity. **C)** Schematic showing current hypotheses as to why responses of VN and VPL neurons are attenuated during active motion. Specifically, the efference copy of the motor command for voluntary movement is used to build an internal model of the expected consequences of movement that is then compared with actual proprioceptive feedback. If there is a match, then a cancellation signal that likely originates within the cerebellum is sent to VN. (For interpretation of the references to colour in this figure legend, the reader is referred to the Web version of this article.)

While recent studies of vestibular thalamocortical neurons have shown important differences in their coding of naturalistic vs. artificial self-motion, vestibular stimuli were passively applied in both conditions. In reality, a major component of self-motion results from voluntary or active movement (e.g., when walking or running) as opposed to passive movement (e.g., when riding a metro) (Carriot et al., 2014). Vestibular afferents respond to head motion signals irrespective of whether these are applied passively or are the result of voluntary movements (Jamali et al., 2009; Cullen and Minor, 2002; Sadeghi et al., 2007; Mackrous et al., 2022). This is not the case, however, for higher brain areas. Specifically, previous studies have shown that neural responses within the brainstem (e.g., vestibular nuclei) are strongly attenuated during active but not passive self-motion (Fig. 4A). As a result, these neurons respond preferentially to the passive component of self-motion when such motion is applied when the animal makes a voluntary head movement (Cullen, 2004, 2011, 2012).

This observation then raises the question of whether vestibular thalamocortical neurons likewise differentially encode active and passive self-motion. A recent study found that indeed the responses of vestibular thalamocortical neurons are greatly attenuated during active self-motion (Fig. 4A) (Dale and Cullen, 2019). Importantly, this attenuation was even more marked than that of vestibular nuclei neurons (Fig. 4B). These findings raise the question: what mechanisms mediate the observed suppression to actively generated vestibular stimulation at the level of the vestibular thalamus? First, the obvious hypothesis that response attenuation is all or none can be rejected since neurons respond to the passive component of self-motion when active and passive self-motion occur together (Dale and Cullen, 2019). Second, attenuation is not directly mediated via inhibitory input resulting from an efference copy of the motor command (von Holst and Mittelstaedt, 1950), as no responses were seen when animals attempted to make head movements but were prevented from doing so via head restraint (Dale and Cullen, 2019). Rather, response attenuation occurs only when there is a match between the predicted and actual consequences of voluntary motion as determined from the efference copy and proprioceptive signals (Brooks and Cullen, 2014; Cullen and Zobeiri, 2021) (Fig. 4C). Further, the fact that neurons in multiple brain areas including the vestibular nuclei display response attenuation to active head on body movements suggests that response attenuation seen in the vestibular thalamus is, at least in part, inherited from these areas. Current evidence suggests that the cancellation signal received by vestibular nuclei neurons during active motion is mediated by the cerebellum (Mackrous et al., 2022; Brooks and Cullen, 2013). Moreover, this inherited response attenuation then appears to be further refined (i.e., enhanced) in vestibular thalamocortical neurons.

Overall, these results have important implications for determining how self-motion perception is generated by multiple cortical areas with vestibular sensitivity such as the parietoinsular vestibular cortex, the ventral intraparietal cortex, area 2v of the intraparietal sulcus, and area 3a in the sulcus centralis that most likely receive input from vestibular thalamocortical neurons (Lopez and Blanke, 2011; Dieterich et al., 2005; Matsuzaki et al., 2004; Akbarian et al., 1994). Indeed, results show that intraparietal cortical neurons display attenuated responses to actively generated self-motion stimuli (Klam and Graf, 2003). Interestingly however, a study reported that neurons within the parieto-insular vestibular cortex do not display response attenuation during active movements (Shinder and Newlands, 2014). It is important to note that vestibular cortical areas also receive input from other modalities (e.g., vision, proprioception, efference copy), which can also provide information about head motion during active movements. Further studies are thus needed to understand how cortical neurons integrate input from vestibular thalamocortical neurons, as well as from extra-vestibular cues, to mediate self-motion perception.

## 6 Conclusion

Results across sensory modalities have progressively overturned the hypothesis that the functional role of the thalamus is to simply relay information from the periphery to the cortex (Mitchell et al., 2014; Wurtz et al., 2011; Bartlett, 2013; Buchan et al., 2021). As reviewed above, a series of recent studies have firmly established that this is also the case for the vestibular system. First, the response properties of these neurons (i.e., how their sensitivity and variability vary with stimulus amplitude) provide a neural correlate for the violations of Weber's law previously reported in human perceptual studies. Second, these same neurons show unambiguous coding of natural but not artificial stimuli that is not simply inherited from afferent input. The lack of phase advance observed in vestibular thalamocortical neurons during natural stimulation is thus thought to be advantageous for perception, while the phase leads displayed by their input vestibular nuclei neurons are instead useful for optimizing motor control. Lastly, they also more selectively encode passive self-motion (Fig. 4). It is important to note that, to date, vestibular thalamocortical neural responses have been primarily measured using yaw rotations and translations along the horizontal plane. Further studies are needed to investigate responses to other motion dimensions (e.g., roll rotations, vertical translations, as well as combinations). Taken together, these recent findings suggest that a critical rethinking of the view that the vestibular thalamus serves as a relay between the vestibular nuclei and cortex during self-motion is necessary. Specifically, the vestibular thalamus plays an essential role in the neural computations that underlie our subjective awareness of motion, as well as our sense of agency of the vestibular consequences of our actions. Further studies are needed to understand how neurons within vestibular cortical areas involved in self-motion perception decode information transmitted by the vestibular thalamus.

## Funding

K.E.C. received funding from the 10.13039/100000002National Institutes of Health (UF1NS111695, R01DC018304, RDC002390, and R01DC018061). M.J.C. and K.E.C. received funding from the 10.13039/501100000024Canadian Institutes of Health Research (CIHR; MOP 162285).

## CRediT authorship contribution statement

**Kathleen E. Cullen:** Conceptualization, Writing – original draft, Writing – review & editing. **Maurice J. Chacron:** Conceptualization, Writing – original draft, Writing – review & editing.

## Declaration of competing interest

The authors declare that they have no known competing financial interests or personal relationships that could have appeared to influence the work reported in this paper.

## Data Availability

No data was used for the research described in the article.
