# Supplementary material for: Neural substrates of perception in the vestibular thalamus during natural self-motion: A review
Source: Curr Res Neurobiol. 2023 Jan 11;4:100073. doi: 10.1016/j.crneur.2023.100073 (PMC10011815; doi:10.1016/j.crneur.2023.100073)
Supplement: Multimedia component 1 [file mmc1.pdf]

Peer Review Overview

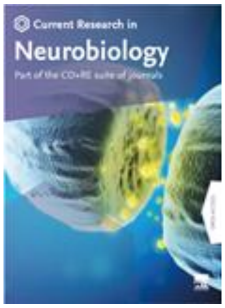

Manuscript Title: Neural substrates of perception in the vestibular thalamus during natural self-motion: a review.

Received	Oct 15, 2022
1st Decision	Nov 18, 2022
1st Revision Submitted	Dec 01, 2022
Accepted	Jan 03, 2023

1st Decision letter

Reference: CRNEUR-D-22-00131
Title: Neural substrates of perception in the vestibular thalamus during natural self-motion: a review.
Journal: Current Research in Neurobiology

Dear Dr. Cullen,

Thank you for submitting your manuscript to Current Research in Neurobiology.

I have completed my evaluation of your manuscript. The reviewers recommend reconsideration of your manuscript following minor revision and modification. I invite you to resubmit your manuscript after addressing the comments below. Please resubmit your revised manuscript by Dec 18, 2022.

When revising your manuscript, please consider all issues mentioned in the reviewers' comments carefully: please outline every change made in response to their comments and provide suitable rebuttals for any comments not addressed. Please note that your revised submission may need to be re-reviewed.

Current Research in Neurobiology values your contribution and I look forward to receiving your revised manuscript.

Kind regards,

Anna S Mitchell, Ph.D.
Editor in Chief
Current Research in Neurobiology

Comments from Editors and Reviewers:

The two reviewers and myself very much enjoyed reading your manuscript. As indicated by the two reviewers, only minor revisions are required to further clarify a very well written and informative review. I look forward to receiving your revised manuscript in due course.

Reviewer #1:

In the current review, the authors summarized recent proceedings in the vestibular research on thalamus, in particular, the ventral posterior lateral part (VPL) that projects to the cerebral cortex. These findings were summarized in three aspects: 1) neural correlates that may mediate the Webb-law deviant behavior; 2) responses to natural vs. artificial (sinusoidal) motion stimulus; and 3) responses to active vs. passive motions. By comparison with the other areas, such as the vestibular nucleus, the VPL in thalamus exhibits different properties that may explain many important psychophysical results. The results were summarized well, and at the same time, they will intrigue future research on some important, albeit not resolved questions in the field. I only have one point that the authors may want to discuss about in the review:
In the third part, based on the attenuated responses during active motion in a number of subcortical areas including VN and VPL, it is natural to infer that responses in the cortex are also expected to be reduced. This hypothesis is supported by the VIP study which has been mentioned in the paper. However, there is also one study showing that the responses in the vestibular cortex (PIVC) largely remain during active motion (Shinder and Newlands, 2014). How this is possible if majority of the responses in VPL have been eliminated by active motion? In addition, if most of the vestibular responses are eliminated by active motion, how perception of the movement status of the head or whole body could be achieved during active motion?

Reviewer #2:

First, the reviewer would like to congratulate the authors. This brief review is extremely well written and above all clear for an average reader despite the difficulties inherent in the subject. Here are some points that can help clarify some concepts described by the authors. The figures are very helpful.

"Specifically, these neurons provide a substrate for previous psychophysical observations that perceptual discrimination thresholds are much better than predictions from,Weber's law."

Many readers of this interesting article will unfortunately only read the summary. In this context, this sentence, while relevant, is nevertheless difficult to understand for a wider audience than aficionados of the vestibular system. Please make it more understandable for the average reader

"It comprises projections from the anterodorsal thalamic nucleus (ADN) to the retrosplenial and entorhinal cortices (Fig. 1, upper right). This pathway has been wellstudied and is thought to provide a neural representation of heading direction during movement that is critical for navigation (see [8,9] for review). The posterior pathway, which is the focus of the current review, involves direct projections from vestibular nuclei neurons to neurons within the ventral posteriolateral area (VPL) of the thalamus that in turn project to vestibular cortical areas (Fig. 1, upper left)."

I think it would be helpful if the reader could refer to a more detailed and realistic diagram than the

current block diagram in Figure 1 to get an idea of the location of the anatomical structures cited in this sentence.

"Vestibular sensitive neurons within VPL,"

Just out of curiosity for the interested reader, please briefly mention the other types of neural processing that take place in the VPL

"The vestibular nuclei neurons projecting to VPL respond to passive sinusoidal rotations around the earth-vertical axis and/or translations in the horizontal plane and are insensitive to eye movements."

It will be helpful to be more specific. What do you mean around the vertical axis of the earth? You mean parallel to the plane of sustentation? For example, what happens when you go down a slope? Also, please explain why in the VPL only neurons responding to rotation of translation in the horizontal plane are shown? Where and how are other representations of space implemented?

"Importantly, neural discrimination thresholds are proportional to the variability-to-gain ratio [26]."

Please explain this sentence in more detail

"Specifically, natural vestibular stimuli reach much higher amplitudes than artificial stimuli and further display spectral power that decays with increasing frequency

Please briefly summarize the numerical values characterizing the upper and lower frequencies of natural vestibular stimulations in human. Maybe a schema will be useful."

"This theory states that information is maximized when the neuronal tuning curve (i.e., the relationship between input stimulus and output firing rate) is the cumulative integral of the stimulus probability distribution [34,39] (Fig. 3C). Sincenatural vestibular stimuli display a bell-shaped probability distribution [32,33,42] (Fig. 3C, left panel, black), the tuning curve that optimizes information transmission has a sigmoidal shape (Fig. 3C, right panel, black)."

This review is crystal clear to an average reader. In this context, I would recommend being more explicit for this particular paragraph, which may be difficult for an untrained reader to understand.

"In contrast, the phase leads displayed by vestibular nuclei neurons during both artificial and naturalistic stimulation as well as those displayed by vestibular
thalamocortical neurons during artificial stimulation led to coding ambiguity."

The way this sentence reads seems like the vestibular nuclei neuron is not doing a proper job. At the other end, one could argue that these neurons are primarily involved in motor control that require increasing phase advance to compensate for increased viscoelastic forces at the periphery. Please comment.

In the conclusion, it might be helpful to highlight the difference between the vestibular networks involved in perception and motor control. As this review shows, the hallmark of the thalamic neurons receiving vestibular input is well suited for perception. At the other end, and this is not mentioned in the

text, the vestibulo-ocular and vestibulo-spinal circuits are optimized for motor control ie to implement the vestibulo-ocular and vestibulo-spinal synergies.

1st Author Response Letter

Response to comments from Editors and Reviewers:

Comments from Reviewer 1

In the current review, the authors summarized recent proceedings in the vestibular research on thalamus, in particular, the ventral posterior lateral part (VPL) that projects to the cerebral cortex. These findings were summarized in three aspects: 1) neural correlates that may mediate the Webb-law deviant behavior; 2) responses to natural vs. artificial (sinusoidal) motion stimulus; and 3) responses to active vs. passive motions. By comparison with the other areas, such as the vestibular nucleus, the VPL in thalamus exhibits different properties that may explain many important psychophysical results. The results were summarized well, and at the same time, they will intrigue future research on some important, albeit not resolved questions in the field. I only have one point that the authors may want to discuss about in the review:
In the third part, based on the attenuated responses during active motion in a number of subcortical areas including VN and VPL, it is natural to infer that responses in the cortex are also expected to be reduced. This hypothesis is supported by the VIP study which has been mentioned in the paper. However, there is also one study showing that the responses in the vestibular cortex (PIVC) largely remain during active motion (Shinder and Newlands, 2014). How this is possible if majority of the responses in VPL have been eliminated by active motion? In addition, if most of the vestibular responses are eliminated by active motion, how perception of the movement status of the head or whole body could be achieved during active motion?

>> We understand the reviewer's and agree that this is an important paper to cite. Given the multi-modal nature of PIVC, we agree that this is an interesting area for future work. We have expanded the text accordingly.

Comments from Reviewer 2

First, the reviewer would like to congratulate the authors. This brief review is extremely well written and above all clear for an average reader despite the difficulties inherent in the subject. Here are some points that can help clarify some concepts described by the authors. The figures are very helpful.

>> We thank the reviewer.

"Specifically, these neurons provide a substrate for previous psychophysical observations that perceptual discrimination thresholds are much better than predictions from Weber's law."

Many readers of this interesting article will unfortunately only read the summary. In this context, this sentence, while relevant, is nevertheless difficult to understand for a wider audience than aficionados of the vestibular system. Please make it more understandable for the average reader

>> We have changed the sentence accordingly.

"It comprises projections from the anterodorsal thalamic nucleus (ADN) to the retrosplenial and entorhinal cortices (Fig. 1, upper right). This pathway has been well studied and is thought to provide a neural representation of heading direction during movement that is critical for navigation (see [8,9] for review). The posterior pathway, which is the focus of the current review, involves direct projections from vestibular nuclei neurons to neurons within the ventral posteriolateral area (VPL) of the thalamus that in turn project to vestibular cortical areas (Fig. 1, upper left)."

I think it would be helpful if the reader could refer to a more detailed and realistic diagram than the current block diagram in Figure 1 to get an idea of the location of the anatomical structures cited in this sentence.

>> We have now included references to previously published more detailed diagrams in the caption of Figure 1. We have also modified Figure 1 to better emphasize that the projection from VN to the ADN is indirect via multiple brain areas.

"Vestibular sensitive neurons within VPL,"

Just out of curiosity for the interested reader, please briefly mention the other types of neural processing that take place in the VPL

>> We now mention that neurons within VPL also respond to proprioceptive as well as somatosensory signals.

"The vestibular nuclei neurons projecting to VPL respond to passive sinusoidal rotations around the earth-vertical axis and/or translations in the horizontal plane and are insensitive to eye movements."

It will be helpful to be more specific. What do you mean around the vertical axis of the earth? You mean parallel to the plane of sustentation? For example, what happens when you go down a slope? Also, please explain why in the VPL only neurons responding to rotation of translation in the horizontal plane are shown? Where and how are other representations of space implemented?

>> We have clarified the sentence to say that, by earth vertical axis, we mean rotations along the yaw axis (i.e., the horizontal canals are roughly parallel to the floor). We now emphasize that previous studies looking at the responses to VPL neurons have primarily used rotations along the yaw axis and/or translations within the horizontal plane. How these neurons respond to vestibular stimuli along other motion dimensions should be the focus of future studies in the conclusion.

"Importantly, neural discrimination thresholds are proportional to the variability-to-gain ratio [26]."

Please explain this sentence in more detail

>> We now explain this sentence in more detail.

"Specifically, natural vestibular stimuli reach much higher amplitudes than artificial stimuli and further

display spectral power that decays with increasing frequency

Please briefly summarize the numerical values characterizing the upper and lower frequencies of natural vestibular stimulations in human. Maybe a schema will be useful."

>> We now mention that the natural frequency range for vestibular stimuli is 0-20 Hz and now point out that the frequency spectra of naturalistic and artificial stimuli are illustrated in the top left and right insets of Fig. 3A.

"This theory states that information is maximized when the neuronal tuning curve (i.e., the relationship between input stimulus and output firing rate) is the cumulative integral of the stimulus probability distribution [34,39] (Fig. 3C). Since natural vestibular stimuli display a bell-shaped probability distribution [32,33,42] (Fig. 3C, left panel, black), the tuning curve that optimizes information transmission has a sigmoidal shape (Fig. 3C, right panel, black)."

This review is crystal clear to an average reader. In this context, I would recommend being more explicit for this particular paragraph, which may be difficult for an untrained reader to understand.

>> We have expanded this paragraph in order to make it more generally accessible.

"In contrast, the phase leads displayed by vestibular nuclei neurons during both artificial and naturalistic stimulation as well as those displayed by vestibular thalamocortical neurons during artificial stimulation led to coding ambiguity."

The way this sentence reads seems like the vestibular nuclei neuron is not doing a proper job. At the other end, one could argue that these neurons are primarily involved in motor control that require increasing phase advance to compensate for increased viscoelastic forces at the periphery. Please comment.

>> We agree and have added a sentence mentioning that the phase leads displayed by vestibular nuclei neurons are thought to help compensate for the inertia of the head-neck system when generating compensatory movements during vestibulo-spinal reflexes.

In the conclusion, it might be helpful to highlight the difference between the vestibular networks involved in perception and motor control. As this review shows, the hallmark of the thalamic neurons receiving vestibular input is well suited for perception. At the other end, and this is not mentioned in the text, the vestibulo-ocular and vestibulo-spinal circuits are optimized for motor control ie to implement the vestibulo-ocular and vestibulo-spinal synergies.

>> We understand the reviewer’s point and now mention these differences in the conclusion.

Accept Letter

Dear Dr. Cullen,

Thank you for submitting your manuscript to Current Research in Neurobiology. Congratulations.

I am pleased to inform you that your manuscript has been accepted for publication.

My comments, and any reviewer comments, are below.

Your accepted manuscript will now be transferred to our production department. We will create a proof which you will be asked to check, and you will also be asked to complete a number of online forms required for publication. If we need additional information from you during the production process, we will contact you directly.

We appreciate and value your contribution to Current Research in Neurobiology. We regularly invite authors of recently published manuscript to participate in the peer review process. If you were not already part of the journal's reviewer pool, you have now been added to it. We look forward to your continued participation in our journal, and we hope you will consider us again for future submissions.

CRNEUR aims to be a unique, community-led journal, as highlighted in the Editorial Introduction. As part of this vision, we will be regularly seeking input from the scientific community and encourage you and your co-authors to take the survey.

We would also like to invite you to take part in our CRNEUR Author Question & Answer (Q&A), which could get published alongside your article and help to promote it. We suspect you might have an interesting story of perseverance or team work that was required for the research study to complete, or a diversity of perspectives that you might share, as a way of inspiring others about neuroscience.

Kind regards,

Anna S Mitchell, Ph.D.
Editor in Chief
Current Research in Neurobiology

Editor and Reviewer comments:

Reviewer 1: The authors have revised the paper well. I have no further questions.

Reviewer 2: The reviewer thank it's the orders to have taken into account all his suggestions. Also being the date I wish to the editors and the orders a very happy Christmas!

-------- *End of Review Comments* --------
